# Novel Adaptive Laser Scanning Method for Point Clouds of Free-Form Objects

**DOI:** 10.3390/s18072239

**Published:** 2018-07-11

**Authors:** Yufu Zang, Bisheng Yang, Fuxun Liang, Xiongwu Xiao

**Affiliations:** 1 School of Remote Sensing & Geomatics Engineering, Nanjing University of Information Science & Technology, Nanjing 210044, China; 2 State Key Laboratory of Information Engineering in Surveying, Mapping, and Remote Sensing, Wuhan University, Wuhan 430079, China; bshyang@whu.edu.cn (B.Y.); liangfuxun@whu.edu.cn (F.L.); xwxiao@whu.edu.cn (X.X.)

**Keywords:** adaptive representation, geometric multi-level, surface variation, radial basis function, perceptual quality

## Abstract

Laser scanners are widely used to collect coordinates, also known as point-clouds, of three-dimensional free-form objects. For creating a solid model from a given point-cloud and transferring the data from the model, features-based optimization of the point-cloud to minimize the number if points in the cloud is required. To solve this problem, existing methods mainly extract significant points based on local surface variation of a predefined level. However, comprehensively describing an object’s geometric information using a predefined level is difficult since an object usually has multiple levels of details. Therefore, we propose a simplification method based on a multi-level strategy that adaptively determines the optimal level of points. For each level, significant points are extracted from the point cloud based on point importance measured by both local surface variation and the distribution of neighboring significant points. Furthermore, the degradation of perceptual quality for each level is evaluated by the adjusted mesh structural distortion measurement to select the optimal level. Experiments are performed to evaluate the effectiveness and applicability of the proposed method, demonstrating a reliable solution to optimize the adaptive laser scanning of point clouds for free-forms objects.

## 1. Introduction

Laser scanning is widely used to rapidly collect the three-dimensional (3D) coordinates of object surfaces for many applications, such as urban planning and cultural heritage protection. However, high density laser scanning point clouds considerably increases the data volume, creating challenges in terms of data storage, processing, visualization, and transmission [1]. Conversely, processing all the points may not provide a significant improvement in the point clouds-based applications (e.g., feature extraction, 3D reconstruction). Hence, simplifying the original point clouds is necessary so that a more manageable dataset can be selected without discarding important features or structures.

Consequently, various approaches have been proposed to tackle the simplification of original point clouds. The existing methods can be classified into three main methods: mesh-based, surface approximation, and direct point simplification.

Based on the tin or tetrahedral mesh of an object, mesh-based methods simplify points by reducing the number of meshes [2,3]. Vertex decimation is one of the approaches used to simplify the mesh [4,5,6] by iteratively removing the least important point and its adjacent mesh. Tseng et al. [7] and Sun et al. [8] applied mesh merging. This approach uses metrics to determine the meshes to be merged or generates a new point to replace all points within a grid. In addition, Shi et al. [9] and Digne et al. [10] constructed an energy function to describe the number, regularity, and quality of points, and simplified the mesh by minimizing the energy. Other, more extended methods include edge contraction [11] and dynamic deformation simplification [12]. In these methods, meshes must be initially constructed. The constructed meshes easily describe the adjacency relationship of points and the local continuous surface. However, the computational and storage costs of mesh construction constitute the disadvantages of these methods.

Various methods simplify points by surface approximation, such as the application of hierarchy strategy. Ohtake et al. [13] fitted hierarchical surfaces based on the sampled point clouds. The interpolation of the coarse surface was used to guide the interpolation of the finer surface until the number of points met the requirement. In contrast, Alexa et al. [14] applied the reverse order of fine-to-coarse in their method. The authors first approximated a smooth surface close to the original using the moving least squares (MLS) method, and then resampled the surface gradually to generate an adequate representation according to the error metric. Local surface fitting methods have also been investigated. Sim et al. [15] partitioned the point cloud by an octree and constructed height fields of each cell to approximate the local surface. Based on the spatial distribution of the points, height fields were resampled to reduce the point cloud. Similarly, normal vectors have been used instead of height information [16,17]. Additionally, adopting global surface fitting based on an implicit function is also a common approach. Carr et al. [18] used polyharmonic splines to fit a surface from point cloud, which is implicitly defined as the zero set of the radial base function (RBF). In the fitting process, a greedy algorithm resulted in simplified points by reducing the RBF centers. Fuhrmann et al. [19] proposed an implicit function defined as the sum of the basis functions to fit a surface. Then, a cleanup procedure was used to reduce the point cloud by erasing the degenerated part of the surface. Similarly, Passion surface [20], Hermite radial basis functions [21], and elliptical splats [22] were introduced. The above methods do not require the construction of accurate meshes, but the topological relations change since most of the resultant points are not original points.

The direct point simplification method avoids the computational cost of mesh construction and retains the topological relations between points, gaining this method increasing attention. Simplification methods based on a clustering algorithm have been reported, such as incremental clustering [23] and hierarchical clustering [24]. Clustering based methods are computationally efficient but have higher simplification errors. Another method for directly simplifying points is particle simulation, which computes new points by moving points on the point-sampled surface according to repelling forces [25]. The progressive splat decimation method [26] was also proposed. This method uses splat geometry in the decimation criteria and error estimates, providing a better quality result than other progressive splat decimators. In addition, the iterative simplification method [27,28] has received attention due to its high accuracy. The iterative methods have improved the simplification accuracy by retaining more points in areas of high curvature. However, blank areas and holes are easily produced in smooth regions. To solve this problem, the density or distribution of neighboring points has been considered [29,30]. These iterative methods used importance evaluation and an error metric to successively reduce points. The importance of each point indicates the amount of local variation information contributing to the geometric shape, which is quantified by local geometric properties, such as chord angle, normal vector, and curvature [31,32,33]. Apart from the above four categories, many other methods have been introduced, such as Voronoi diagrams and spatial-grids-based methods [34,35]. Furthermore, the field of two-dimensional (2D) image reduction has progressed [36]. Based on this concept, Arnold et al. [37] and Cheng et al. [38] proposed point cloud simplification methods based on image processing. Similarly, Hyun et al. [39] and Cao et al. [40] proposed simplification methods based on analytical equation and image mapping. For objects with flat surfaces, these methods perform well. However, for the objects with complex surfaces, the results are not satisfactory, since large errors are introduced after mapping.

Although the reported iterative simplification methods can generally provide satisfactory results for various point cloud-based applications, they still have limitations. A 3D object usually has multiple geometric features at different levels, called Levels-of-Detail. These multiple levels of features together constitute complete information for an object [41]. However, the reported methods struggle to comprehensively preserve the features because the importance evaluation is performed at a single predefined level.

However, an increasing number of applications are focusing on the perceptual quality of simplified points. Most existing simplification methods are based on geometric error metrics, such as Hough distance, curvature difference, and quadric errors. Therefore, the simplified results mainly describe geometric quality and ignore the visual perception quality. To improve the visual quality, the perceptual metric has been proposed and widely used. For a 3D object, visual masking is an important visual characteristic, which means some geometric features are difficult to perceive, since they are affected by surrounding information [42]. Torkhani et al. [43] proposed a roughness-based weighting of the local tensor distance for visual masking. Shi et al. [44] defined the salient information distortion metric by combining Just Noticeable Difference (JND) with information theory to evaluate the data. The mesh structural distortion measure (MSDM) proposed by Lavoué et al. [45] effectively measured the quality in visual perception. However, this metric is only adaptable to point clouds with identical numbers of points.

To overcome the above two drawbacks, this paper proposes a multi-level approach to reserve geometric features of different sizes, and applies a perceptual metric to measure the visual degradation of the geometric information of each level, thus achieving level-adaptive simplification of the original points for high perceptual quality representation of 3D objects. The main contributions of the proposed method are as follows: (1) we evaluate the importance of each point by 3D Gaussian smoothing and compactly supported radial basis function (CSRBF). This method describes the multiple levels of features and avoids the dense distribution of points and blank areas in the point cloud. (2) We implement a multi-level strategy to avoid the inaccuracy of using a single level, and we apply a perceptual metric to ensure the high perceptual quality of simplification. The combination of these two features means the method is both flexible and adaptable.

The remainder of the paper is structured as follows. Section 2 provides information about the instruments and data acquisition. Section 3 elaborates the key steps of the proposed method, including the surface variation calculation of the 3D points, the construction of multi-level points based on compactly supported radial basis function (CSRBF), and the selection of optimal level based on a perceptual metric. The experiments and evaluations undertaken to demonstrate the validities and advantages of the proposed method are outlined in Section 4. Finally, the conclusions are drawn in Section 5.

## 2. Instruments and Data Acquisition

### 2.1. Sensors

To verify the adaptability and flexibility of the proposed method, four datasets with different geometric characteristics scanned by different laser scanners (e.g., Handyscan 3D (Creaform, Shanghai, China), Rigel VZ 400 (RIEGL, Horn, Austria), and Faro Focus X330 (FARO, Groveport, OH, USA)) were utilized. The specifications of these scanners are listed in Table 1.

### 2.2. Measurements and Experimental Data

Data I are the point clouds of statues from the No. 159 cave in DunHuang MoGao grottoes, scanned by a Handyscan 3D in 2012. Small artificial targets were used to register scanning points of each moment, and point clouds for each station were registered automatically by self-developed software. The measurement lasted for 20 days and 3.6 million points were captured. The average span of points was about 0.8 mm. Data II and data III are the Bologna dataset [46] and car model data provided in http://www.vehiclescans.com/resources. They were scanned by a Synthetic and Faro Focus X330, respectively. Data IV are the point clouds of an indoor scene in Wuhan University scanned by a Rigel VZ400 in March 2018. For each station, we collected an overview of the point clouds. A total of 16 stations were set up and 27.9 million points were scanned. The average span of the point clouds was about 1.0 cm.

**Figure 1:**
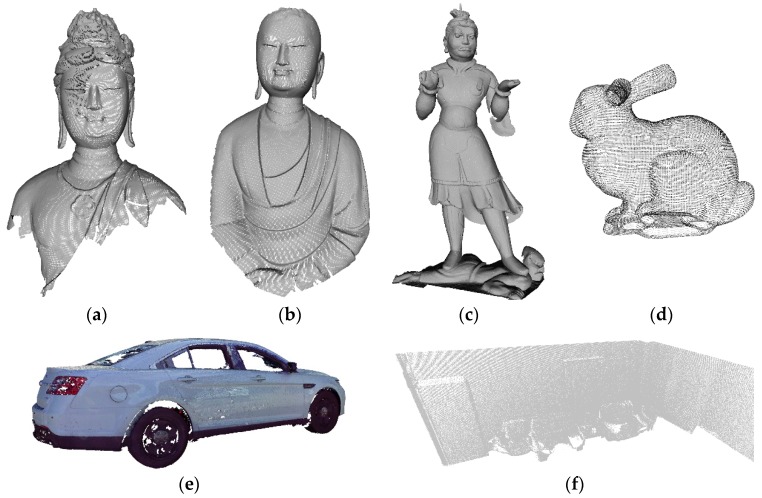
The point clouds from MoGao grottoes and Bologna dataset: (**a**) Bodhisattva model, (**b**) Ananda model, (**c**) general model, (**d**) rabbit model, (**e**) car model, and (**f**) indoor data.

In this paper, partial datasets were used (e.g., data for three statues from Data I, rabbit data from Data II, partial car model from Data III, and partial indoor data from Data IV), which provided sufficient information to verify the performance of this method. The experimental point clouds are shown in Figure 1. The detailed parameters are listed in Table 2.

## 3. Methodology

**Figure 2:**
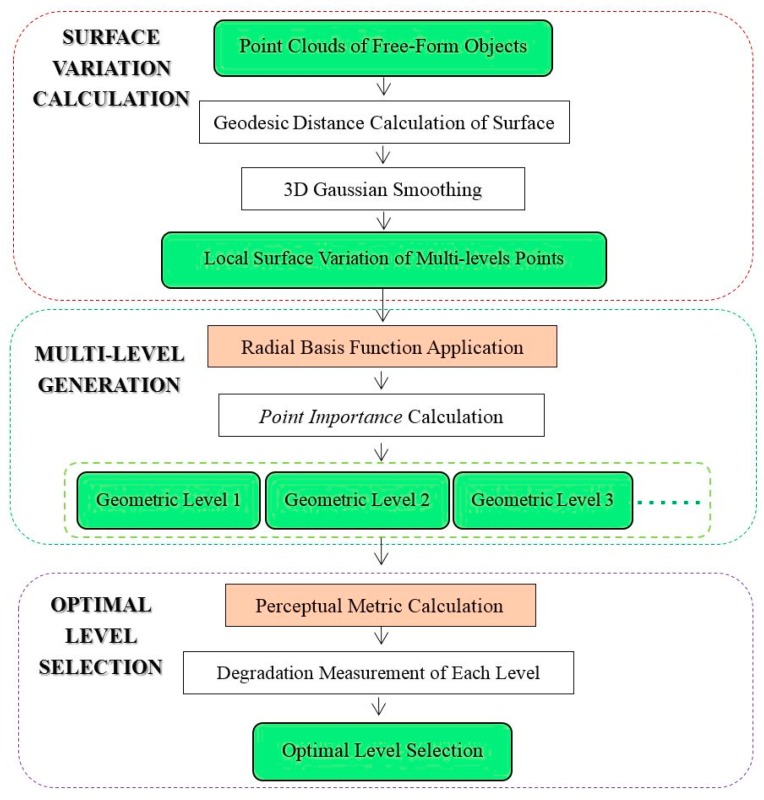
Working flowchart of the proposed method.

The proposed method involves three key components: surface variation calculation based on 3D Gaussian smoothing, importance evaluation by compactly supported radial basis function (CSRBF) and multi-level generation, and optimal level selection according to a perceptual metric. Figure 2 illustrates the framework of the proposed method.

### 3.1. Surface Variation of 3D Surface Points

Surface variation indicates the degree of geometric change in the local surface, encoding local geometric information, which is an important factor in evaluating the point importance of different levels. We define a surface variation metric in a level-dependent manner based on 3D Gaussian kernel. For a point cloud M, *P_v_* is one point from M, the surface variation of *P_v_* is calculated by:

(1) $$S_{v}=\left|P_{v}\;-\;\frac{\sum_{i=1}^{N}P_{i}G(u,v;\delta )}{\sum_{i=1}^{N}G(u,v;\delta )}\right|$$

where *N* is the number of neighborhood points of *P_v_*, *P_i_* indicates one neighborhood point, G(u,v;δ) is the 3D Gaussian kernel, and *Sv* indicates the deviation between point *P_v_* and the new point obtained after Gaussian smoothing.

According to Equation (1), Gaussian smoothing is used to reduce the property deviations between one point and its neighbors to obtain a new smoothed point. To calculate the surface variation in surface points, we define 3D Gaussian kernel for a 3D object as:

(2) $$G(u,v;\delta )=\frac{1}{2\pi \delta^{2}}\exp\left[-\frac{1}{2\delta^{2}}d{\left(u,v\right)}^{2}\right]$$

where *d*(*u*,*v*) is the distance from the current point *v* to one neighborhood point *u*, δ denotes the root mean square error of Gaussian kernel. The larger the mean square root error, the larger the smoothing region, so more significantly visual features will be detected. Hence, the significant visual features at different spatial extents can be detected by adjusting the mean square root error.

**Figure 3:**
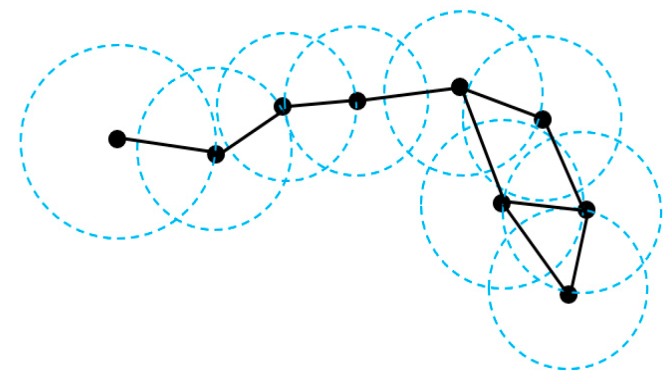
Example of a Sphere-of-Influence graph (SIG).

To accurately construct a geometric scale-space that reliably encodes the surface geometry, the distance *d*(*u*,*v*) in a Gaussian kernel is defined in terms of geodesic distance rather than Euclidean distance. Let *V* = {*p*_1_, …, *p_n_*} be the set of points. The influence sphere of *p_i_* is defined with the center of *p_i_* and the radius as the distance from *p_i_* to its nearest neighbor. The Sphere-of-Influence graph (SIG) is a graph in which two points *p_i_* and *p_j_* are connected by an edge *e_ij_* if the corresponding influence spheres intersect. Each edge is weighted by the Euclidean distance between the connected points. Figure 3 shows a SIG constructed by nine points.

**Figure 4:**
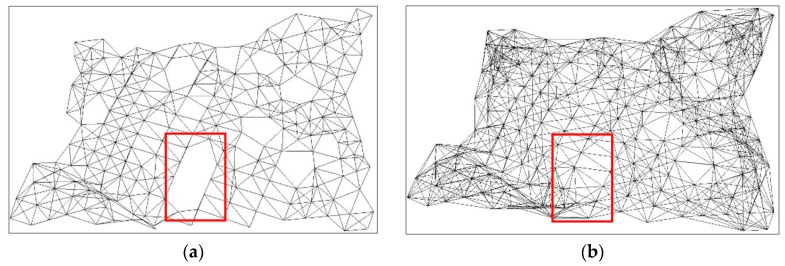
SIGs constructed using: (**a**) the original method and (**b**) our method.

However, blanks may appear in a SIG because the radius of each point is difficult to specify, as the red box illustrates in Figure 4a. Klein et al. [47] set the radius as the distance to the *k*th nearest neighbor with *k* > 1. However, a larger *k* value generates longer edges, leading to less accurate geodesic distance. To solve the problem, the radius of each point is defined according to the point density of local area, written as:

(3) $$r=L\sqrt{\frac{\pi }{Num}}$$

where *Num* is the number of neighborhood points and *L* is the longest distance between two neighborhood points. Figure 4 shows the SIGs constructed using different methods. Given the constructed SIG, the geodesic distance between two points on the object surface can be calculated by the shortest path search algorithm [48].

**Figure 5:**
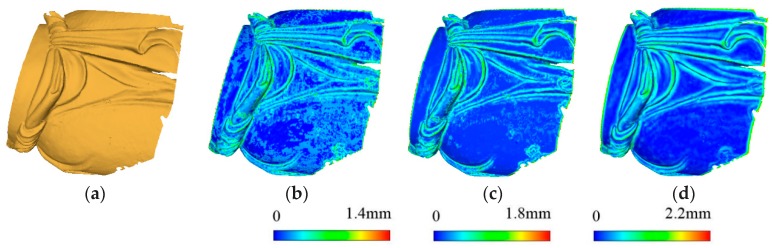
Rendering of surface variations of different levels. (**a**) Original data, (**b**) rendering of surface variations at level δ=2.0, (**c**) rendering of surface variations at level , and (**d**) rendering of surface variations at level δ=4.0.

Figure 5 illustrates the rendering of surface variations with different neighborhood ranges of a small point cloud. The figure demonstrates that the different level features (granularities) are well described by the surface variations calculated by 3D Gaussian smoothing.

### 3.2. Multi-Level Points Generation Based on Degree of Importance

Point importance represents the significance of each point and is used to determine which points should be retained in different levels. To assign the points to different levels, a significance metric called the Degree of Importance (DOI) of points is defined by integrating the surface variation $S_{newi}$ and the distribution of neighborhood significant points, calculated as:

(4) $$\left\{ \begin{array}{l} DOI_{i}=\frac{S_{newi}}{\varepsilon +W\sum_{j=1}^{N}\varphi \left(\|P_{j}-P_{i}\|/\lambda \right)}\\ \varphi (r)={\left(1-r\right)}_{+}^{4}(1+4r)=\left\{ \begin{array}{ll} {\left(1-r\right)}^{4}(1+4r),\; & if\;1>r\\ 0, & if\;1\leq r \end{array}\right. \end{array}\right.$$

where *N* is the number of neighborhood significant points of *P_i_*, *P_j_* is one neighborhood significant point. φ(r) is a compactly supported radial basis function, λ is the support radius factor, *r* indicates the valid supporting region, ε is a constant, and *W* is the weight coefficient, controlling the degree of neighborhood influence. In Equation (4), the application of the radial basis function controls the influence of neighborhood points on the current point. To reduce the calculation burden, the CSRBF introduced by Wendland [49] is applied, and φ(r) with C^2^ continuity is used since the quality with order C^2^ is better than other continuities.

For one point in the feature area, the denominator of Equation (4) is larger since it has more neighborhood feature points, and its DOI decreases and the probability of being removed increases. For points in flat areas, the DOI is the opposite. Therefore, for the simplified point cloud, the dense distribution of points in the feature area and the blanks in the flat area are avoided. This is consistent with the visual masking effect, ensuring high perceptual quality of the simplification. However, the significance metric DOI is level-dependent. The DOI of each point of different levels can be calculated by changing the neighborhood range of variables in Equation (4). This suggests that multiple levels of points can be generated.

However, noise points have larger surface variation values, which are reserved as feature points since they have larger DOI values. This method suppresses the noise by adjusting the surface variation values. The adjusted surface variation $S_{newi}$ after suppression is defined as:

(5) $$S_{newi}=\left\{ \begin{array}{l} \;0\;,\;if\;S_{i}\geq \mu_{S}+3\sigma_{S}\\ \;S_{i},\;if\;S_{i}\;<\mu_{S}+3\sigma_{S} \end{array}\right.$$

where $S_{i}$ is the surface variation of point *P_i_*, $\mu_{S}$ is the mean value, and $\sigma_{S}$ is the mean square error of surface variations of the neighboring points. After suppression, we obtain the DOI value of each point according to Equation (4).

Multi-level points have different sized features, representing different simplification results with different levels of details of an object. As such, the following steps are executed to generate the significant points of multi-level from coarse to fine levels.

**Step** **1.** According to Equations (1) and (5), calculate the surface variation of each point based on the neighborhood range of the current level, and rank all points in decreasing order according to their surface variation values.
**Step** **2.** Select the surface variation value of the point located at the 80% position as the DOI threshold.
**Step** **3.** Take the point from the sorted point set in turn to a destination point set. The original surface variation value of this point and the new neighbors in the supporting region of the destination point set are integrated to calculate its DOI value using Equation (4). If the DOI is larger than the threshold, reserve it; otherwise, remove it from the destination point set.
**Step** **4.** Repeat Step 3 until all the points are processed.
**Step** **5.** Remove the points already existed in the coarser levels.

**Figure 6:**
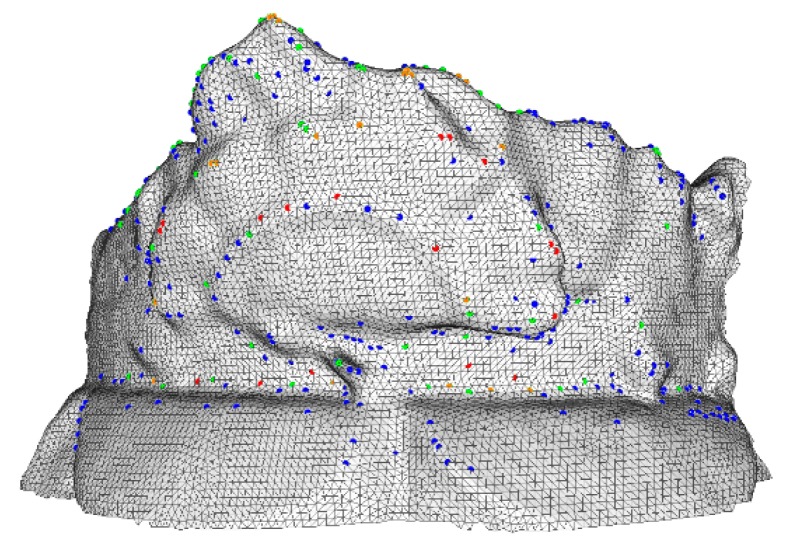
Significant points detected of four levels.

Figure 6 shows the detected significant points of the four levels. Blue, green, orange, and red dots indicate the points obtained from the coarse to fine level. Note that the points are well dispersed across levels and at the correct corresponding levels. This suggests that the significant features of different levels are well described.

**Figure 7:**
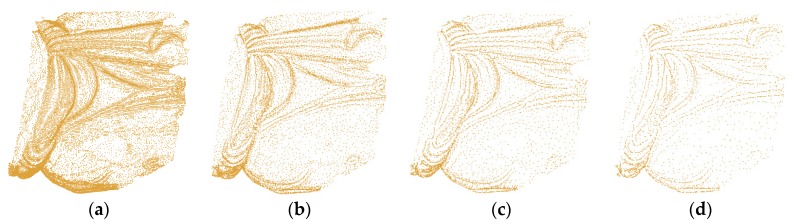
Four levels of points of a small point cloud: (**a**) δ=3.0, (**b**) δ=4.0; (**c**) δ=5.0, and (**d**) *δ* = 6.0.

After the significant points of all levels are determined, merge the points of the current level with the points of all the coarser levels as the final point set of the current level. Figure 7 shows four levels of points of a small point cloud.

### 3.3. Perceptual Metric of Multi-Level and Optimal Level Selection

Different from the quality measured using geometric error metrics, perceptual quality depicts the quality degradation in visual perception. Various applications focus on the perceptual quality of simplified points. The mesh structural distortion measure (MSDM) method proposed by Lavoué et al. [45] effectively evaluates the perceptual quality of 3D point clouds.

**Figure 8:**
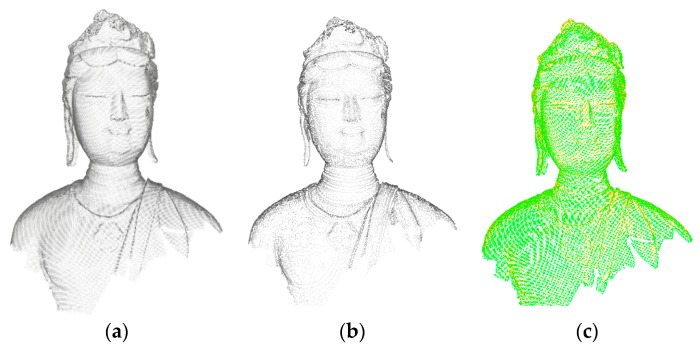
(**a**) Original points, (**b**) point clouds at one level, and (**c**) supplementing the missing points. Orange points are level points and green points are the supplemented points.

However, MSDM cannot be directly used to measure the degradation of different levels since the levels have different number of points. To overcome this drawback, this paper projects the missing original points onto the patch constructed by the three nearest points from the corresponding level, resulting in an identical number of points as the original points at each level. Figure 8 shows the supplement result for the missing points. To improve the accuracy of the degradation measurement based on the neighborhood range of a single level, we calculate the curvatures of each point with the neighborhood ranges at multiple levels to determine its intrinsic scale [35] as the final neighborhood range. Hence, during the calculation of MSDM, the neighborhood range of each point is determined by its own intrinsic property.

Based on the above improvements, the perceptual degradation of one level is calculated by:

(6) $$MSDM(u,v)={\left[\frac{1}{N}\sum_{i=1}^{N}\alpha L{\left(u_{i},v_{i}\right)}^{t}+\beta C{\left(u_{i},v_{i}\right)}^{t}+\gamma S{\left(u_{i},v_{i}\right)}^{t}\right]}^{\frac{1}{t}}$$

where *N* is the number of original point cloud; α,β,γ are constants, α+β+γ=1; *t* is a constant ranging between 2.5 and 4.0; *L*, *C*, and *S* are the curvature difference, contrast difference, structure difference between the points of current level and the original point clouds, respectively. For one original point *u* and its corresponding point *v*, *L*, *C*, and *S* in Equation (6) are calculated by:

(7) $$L(u,v)=\frac{\left|c_{u}-c_{v}\right|}{\max(c_{u},c_{v})},C(u,v)=\frac{\left|\sigma_{u}-\sigma_{v}\right|}{\max(\sigma_{u},\sigma_{v})},S(u,v)=\frac{\left|\sigma_{u}\sigma_{v}-\sigma_{uv}\right|}{\sigma_{u}\sigma_{v}}$$

where $c_{u},c_{v},\sigma_{u},\sigma_{v},\sigma_{uv}$ are the local mean curvatures, standard deviations, and covariance of point *u* and *v*, respectively. The deviations between the original point cloud and the points of each level are measured by *L*, *C*, and *S*. In feature regions, both the local mean curvature and the standard deviation are larger, resulting in a decrease in the perceptual degradation according to Equation (6). This aligns with the 3D visual masking effect.

**Figure 9:**
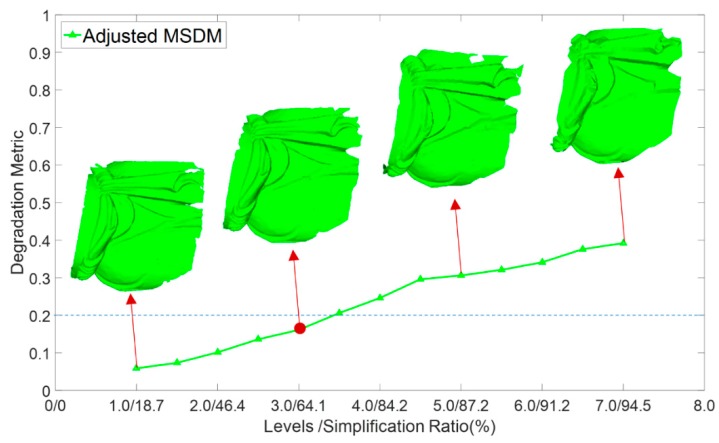
Optimal level selection of small point cloud.

According to the above calculation, the perceptual degradation of each level is obtained. For multiple levels of points, the coarsest level with the MSDM value smaller than the perceptual degradation threshold is selected as the optimal level. Subjective experimental studies suggest that the degradation of the final level can be accepted if the degradation threshold is set between 0.2 and 0.3. In this paper, we used 0.2 as the threshold. As Figure 9 shows, level *δ* = 3.0 is the coarsest level below 0.2, so level δ=3.0 was selected as the optimal level.

## 4. Results and Discussion

The validity and robustness of the multi-level generation was verified by a series of experiments. The implementation details are introduced in Section 4.1 and Section 4.3. In addition, the influence of parameters are also discussed. The performance of the optimal level selection is presented in Section 4.4. We compare the results with three commonly used simplification methods: clustering [50], curvature-based [51], and Poisson-disk [52], demonstrating the reliability and applicability of this method. In this paper, the point cloud was processed by a self-developed software programmed in Visual Studio 2015. For better visualization, the simplified points were triangulated with the software Geomagic studio 2012 and shown on Meshlab 1.3.2.0.

### 4.1. Results and Analysis of Multi-Level Generation

**Figure 10:**
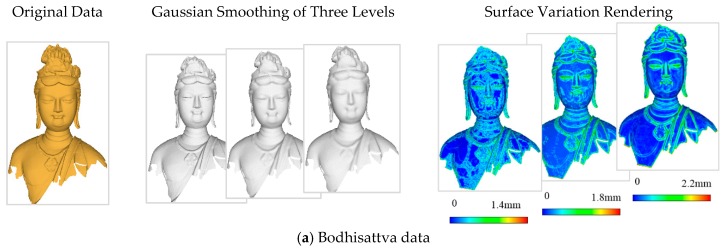

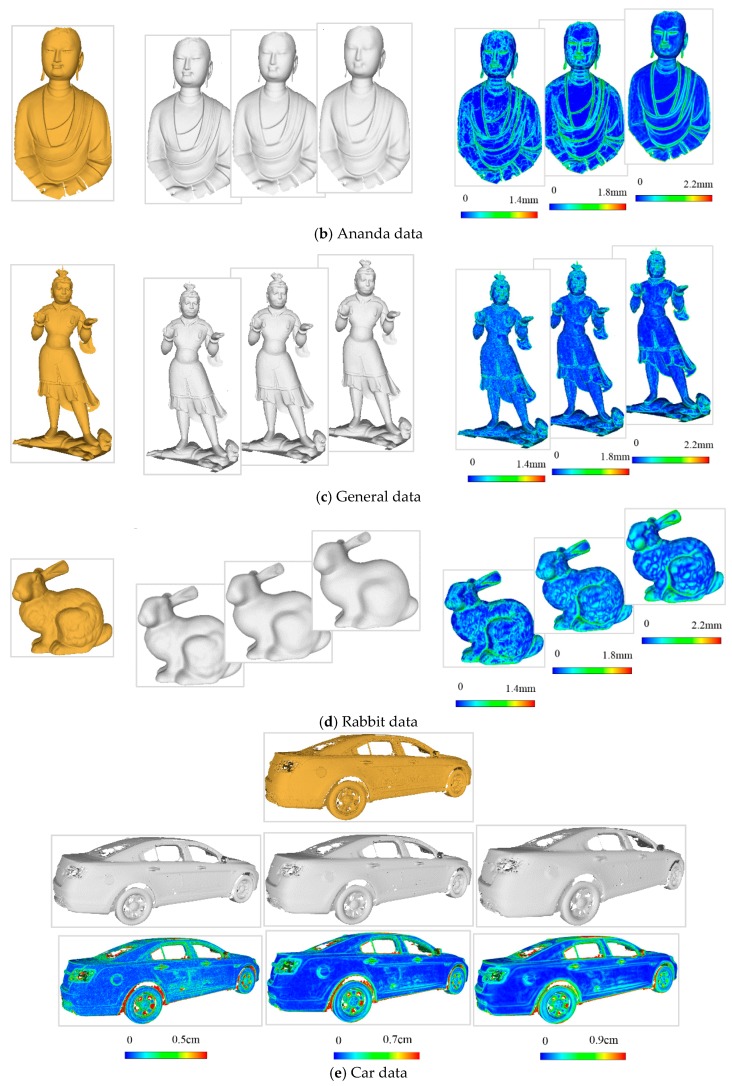

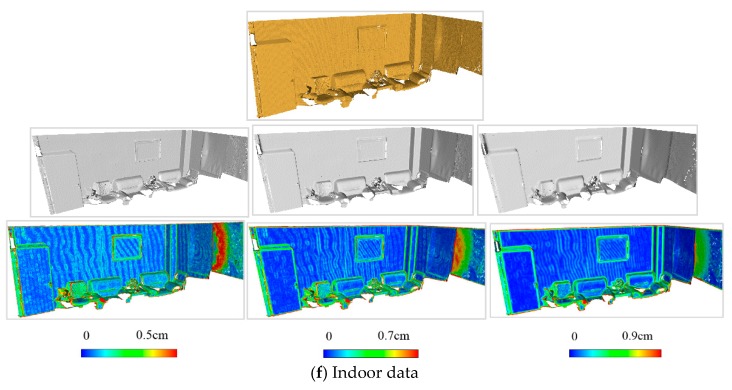
Gaussian smoothing results of three levels and corresponding surface variation rendering.

Figure 10 illustrates the Gaussian smoothing results of the experimental data and the corresponding rendering results of surface variations. Second columns of Figure 10 are the results smoothed by the 3D Gaussian kernel based on geodesic distances at levels of *δ* = 2.0, 4.0, and 6.0. Different levels of geometric features are filtered, encoding the geometric characteristics that can be used to calculate the level-dependent surface variation. The surface variations of different levels, as illustrated in the last columns of Figure 10, illustrate that the geometric features at different spatial extents are well described, indicating that dividing and selecting the critical points of different levels was reasonable.

**Figure 11:**
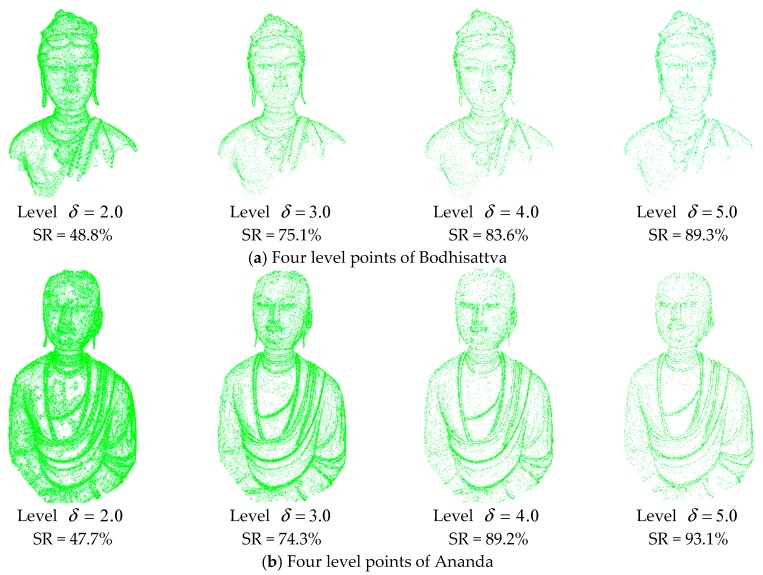

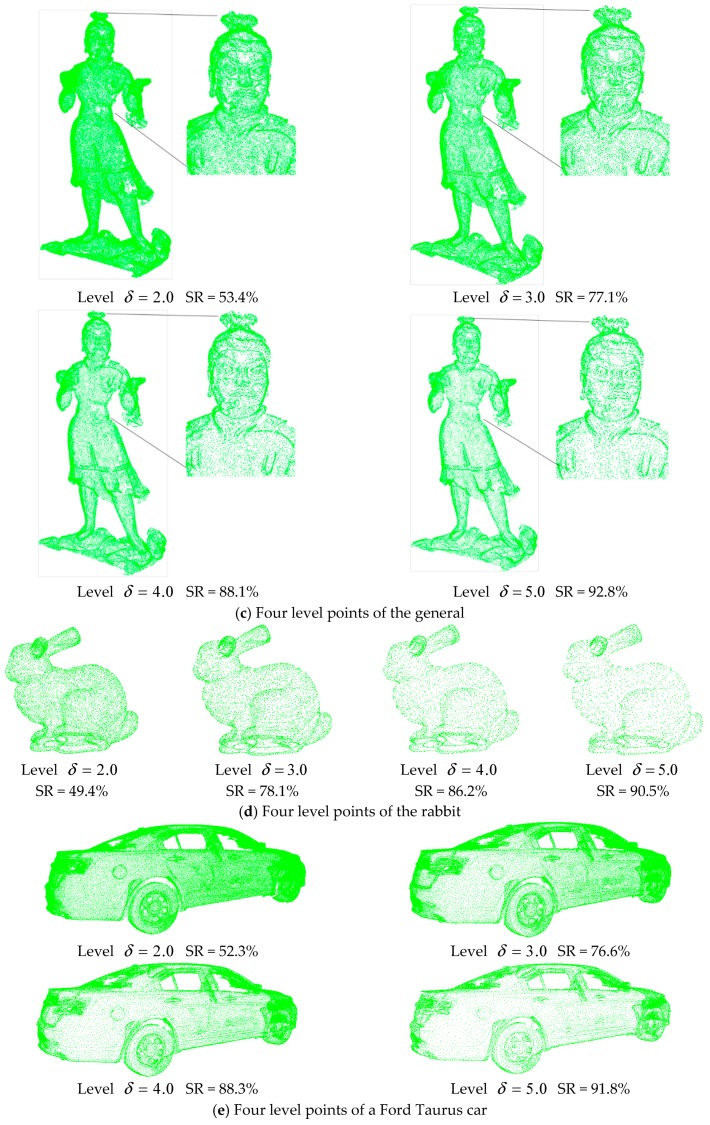

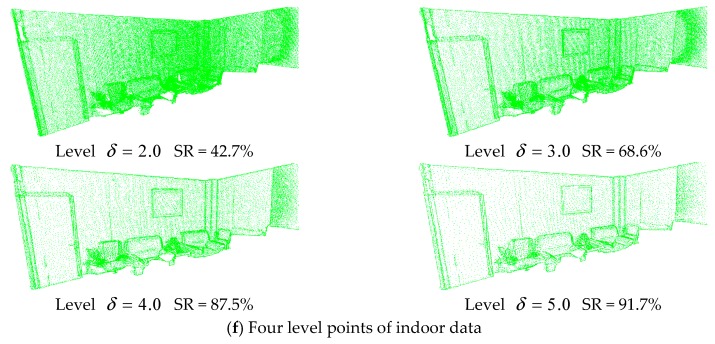
Multi-level points of experimental data.

According to the multi-level points generation method introduced in Section 3.2, we selected points from levels *δ* = 2.0, 3.0, 4.0, and 5.0 based on experimental data. The four levels of points are illustrated in Figure 11, where SR is the point simplification ratio. For one level, the length covered by three times the mean square error of the Gaussian kernel function is often used as the neighborhood radius for the importance measurement. If the radius of one level is determined, the neighborhood ranges of other levels can be calculated according to the proportions between mean square errors. In our implementation, the neighborhood radius of level from level *δ* = 1.0 was set as 1.5 times the average point span (e.g., 1.2 mm for MoGao grottoes data, 1.5 mm for Bologna data, and 1.5 cm for car model and indoor data), so that the multi-level points involve the geometric details at common spatial extents.

**Figure 12:**
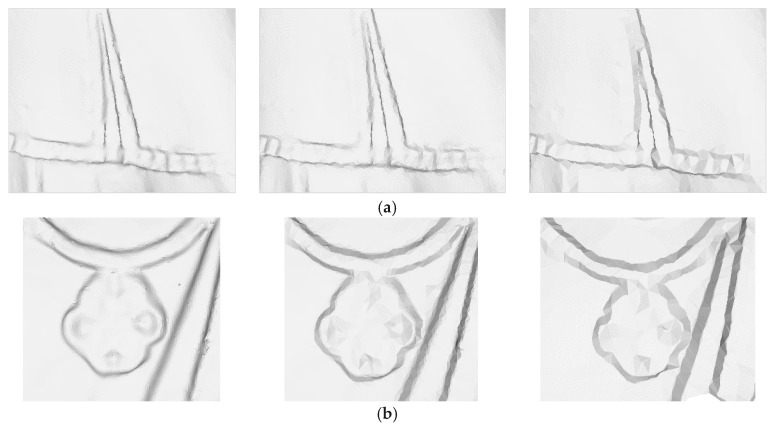
Comparison between 3D meshes at levels *δ* = 1.0, 2.0, and 3.0. (**a**) Multiple levels of smocking, (**b**) Multiple levels of necklace.

Figure 11 illustrates that the selected points at each level accurately describe the features with a high simplification ratio and effectively reduce the data redundancy. The simplification results avoid the overcrowding of points in the feature area and the blanks of points in flat area. This is due to the validity of the significance metric, DOI. In addition, the figure illustrates that the simplified results are determined by the distribution characteristic of the multi-level features, not the geometric sizes or point span of data. Different levels omit different spatial sizes of geometric features, providing a good base for optimal level selection based on the perceptual metric. Particularly, the geometric details at each level are well preserved as illustrated in Figure 12.

### 4.2. Influence of Parameters of Multi-Level Point Generation

**Figure 13:**
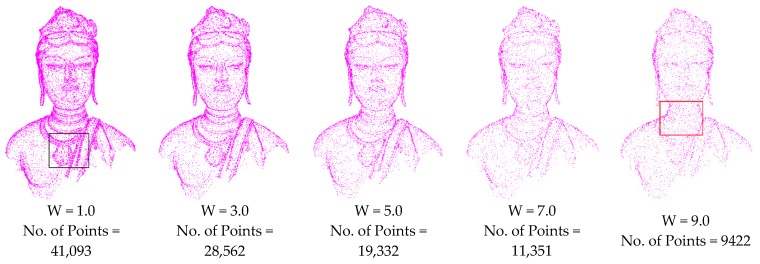
Results of level δ=3.0 with different weight coefficients (λ= 4.0).

**Figure 14:**
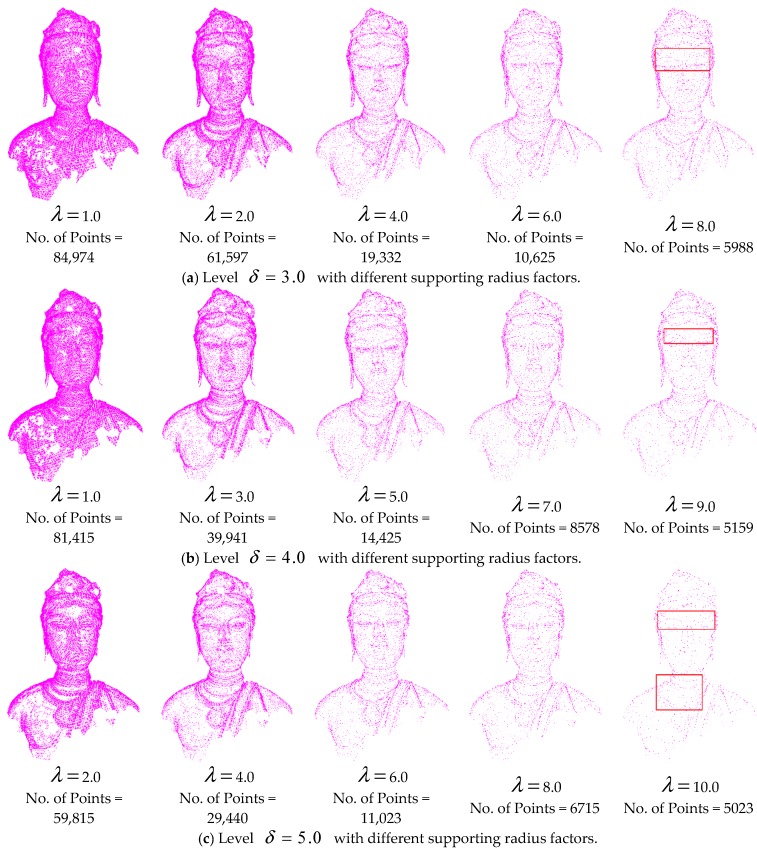
Levels with different supporting radius factors (*W* = 5.0).

From the DOI calculation in Equation (4), the weight coefficient *W* and supporting radius factor λ affect the selection of points for each level. *W* affects the total weight of the contribution of points in the supporting region; λ decides the region of the involved points and the contribution of each point. To investigate the effects of the parameter values, experiments with different parameters values were analysed based on Bodhisattva data, as illustrated in Figure 13 and Figure 14. Figure 13 illustrates five results at level δ=3.0 with different weight coefficients *W*. Figure 14 illustrates the results at levels δ= 3.0, 4.0, and 5.0 with different supporting radius factors λ.

Figure 13 illustrates that the total weight of the points in the supporting region is proportional to weight coefficient *W*. A smaller weight coefficient *W* leads to a larger DOI value, resulting in more points as shown by the black box. However, too large a weight coefficient *W* (e.g., 9.0) leads to sparse points in the feature region, resulting in serious geometric information loss. A visual check determined that *W* = 5.0 is better for the DOI calculation of points.

Figure 14 illustrates the selection of points with different supporting radius factors with the weight coefficient *W* = 5.0. A smaller supporting radius factor leads to larger DOI values of the points, resulting in more points at each level, and vice versa. In addition, the supporting radius factors at three levels of 4.0, 5.0, and 6.0 produce better results. According to Section 3.2, the neighborhood ranges of the points at levels *δ* = 3.0, 4.0, and 5.0 are 3.6, 4.8, and 6.0 mm, respectively. Hence, the supporting radius factor λ for each level is specified as the corresponding neighborhood range, ensuring high perceptual quality and simplification ratio of the simplified points.

### 4.3. Robustness to Noise

**Figure 15:**
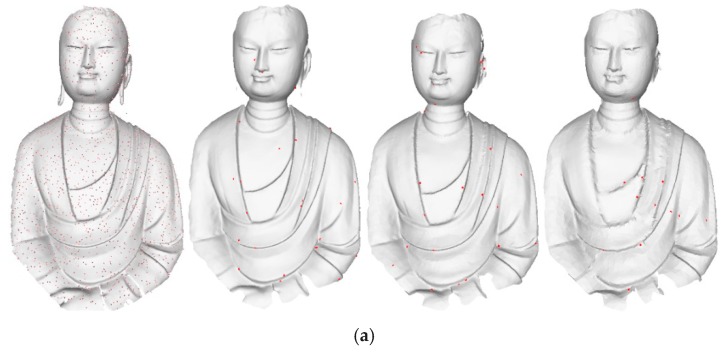

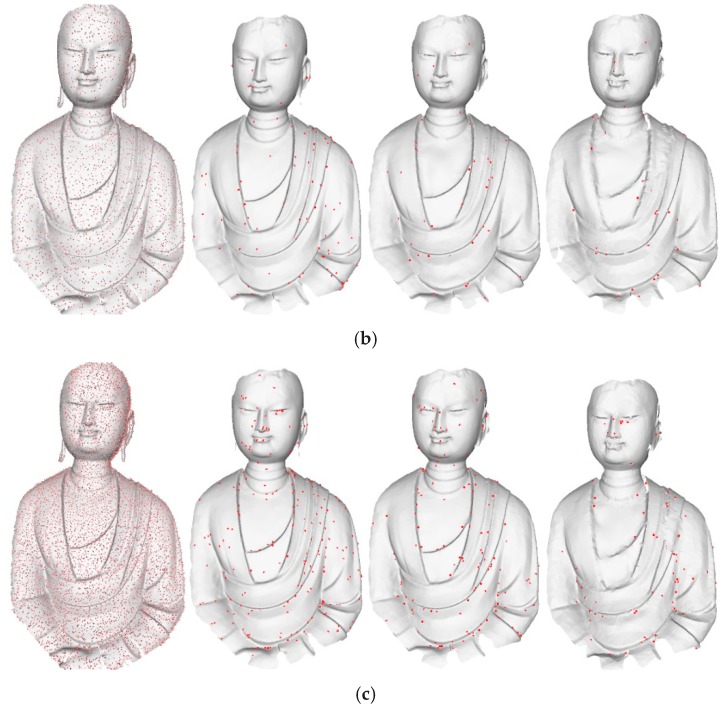
Noisy data and simplified results of level *δ* = 2.0, 4.0, and 6.0 when (**a**) 1% of points are noise points, (**b**) 2% are noise points, and (**c**) and 5% are noise points.

To check the robustness of the proposed method against noisy data, the scanned points of one statue were mixed with noise along the normal direction of the original points. The magnitude of each noise was randomly selected within three times the average point span. Different numbers (1%, 2%, and 5% of the statue point number) of noise points were superimposed on the statue points. For better visualization, the superimposed noises are presented on meshes constructed by original points (Figure 15), and the remaining noise is presented on meshes at level *δ* = 2.0, 4.0, and 6.0. Several indicators were used for quantitative evaluation. The results are listed in Table 3. *No.* is the number of remaining noise points and *M* and *V* are the Mean and Root Mean Square of distances from the remaining noises to the original mesh, respectively.

From Figure 15, the meshes constructed based on simplified points still have high perceptual quality. This demonstrates that noise has minimal impact on the simplification; the proposed method performs well in terms of robustness. Table 3 shows that the mean and root mean square of deviations of remaining noises increase with increasing level. This occurs because, for coarser levels, the surface variations in points are larger, leading to the reservation of noise points with larger magnitude. However, for coarser levels, the reserved noise points have little impact on quality. Conversely, the number of remaining noise points increase as more noise points are added, but still less than 2.5% of total noise points are added. Besides, the mean values and root mean squares do not increase, illustrating that the noise points are well suppressed.

### 4.4. Analysis of Optimal Level Selection and Comparison with Other Methods

**Figure 16:**
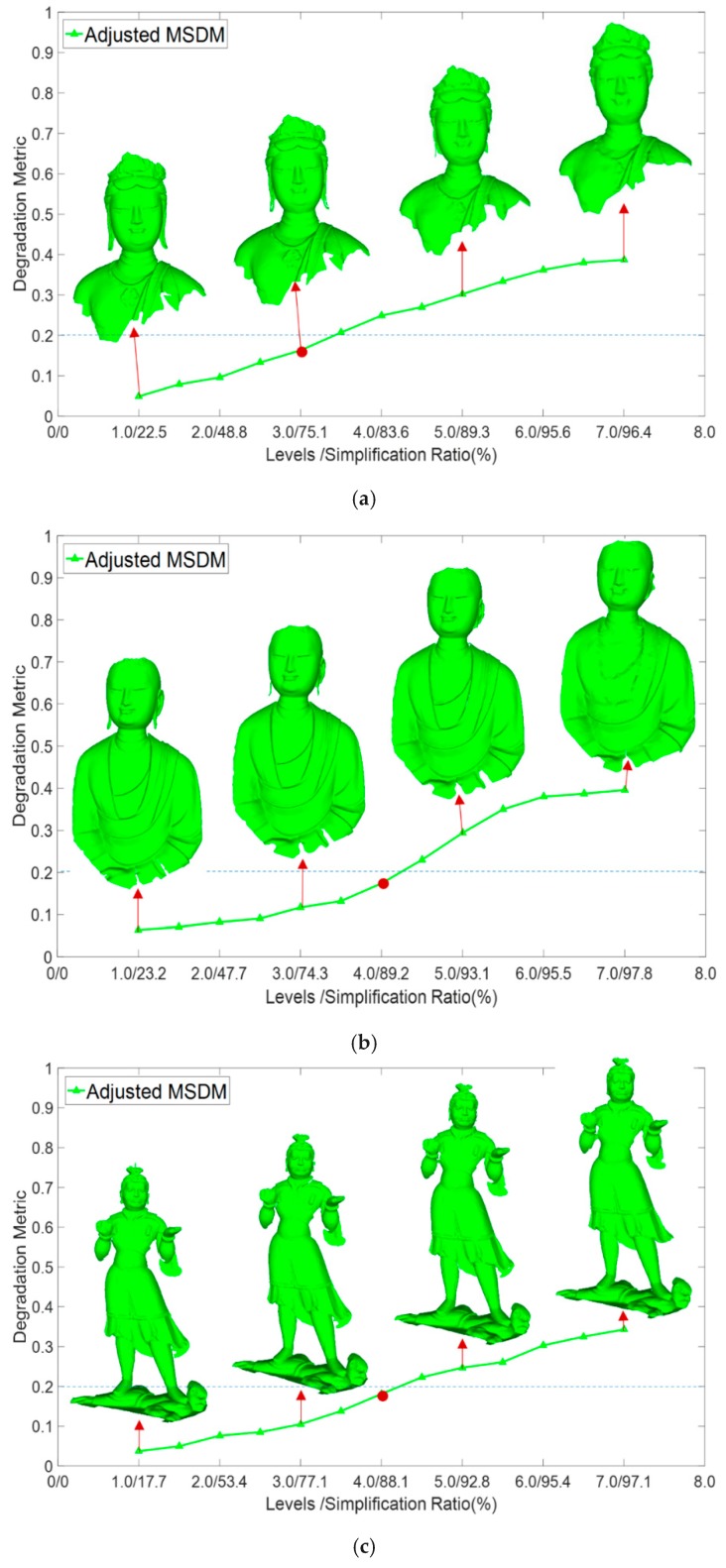

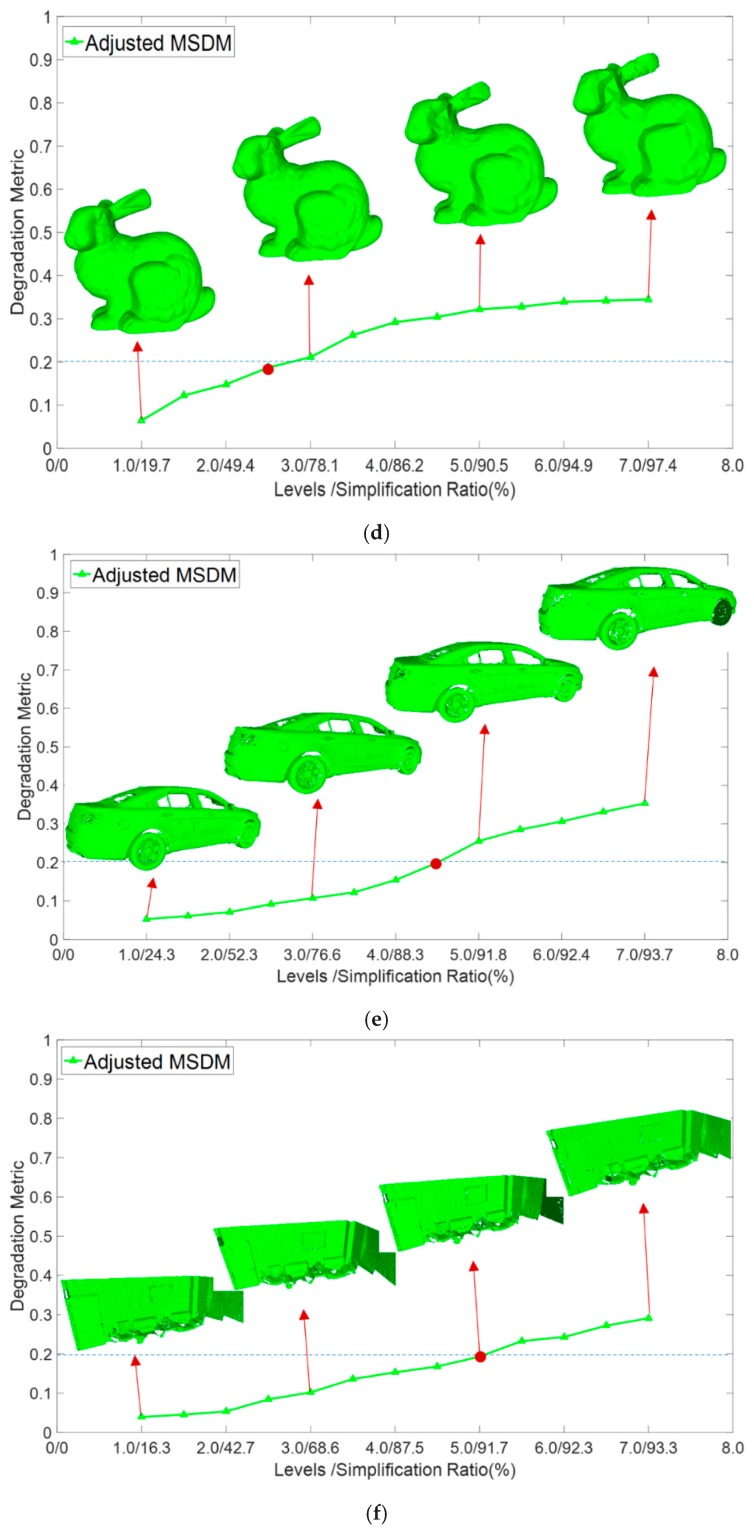
Optimal level selection results. (**a**) Bodhisattva data’s level selection, (**b**) Ananda data’s level selection, (**c**) General data’s level selection, (**d**) Rabbit data’s level selection, (**e**) Car data’s level selection, (**f**) Indoor data’s level selection.

Figure 16 illustrates the results of optimal level selection on the experimental datasets. According to Section 3.3., we evaluated the perceptual degradation values of multiple levels from level δ=1.0 to level δ=7.0. The optimal level was selected based on an acceptable perceptual threshold determined by subjective experiments. In each figure, the vertical axis indicates the MSDM degradation of each level, and the simplification ratio of each level is illustrated on the horizontal axis. The blue dashed line shows the perceptual threshold and the red dot indicates the optimal level.

From Figure 16, for the object with few small-sized features, the perceptual quality of the first few levels degrades slightly (e.g., level *δ* = 1.0–3.0 of Ananda, General, car, and indoor data is within 0.1), leading to a higher optimal level. Otherwise, the degradation change in first few levels is fast, leading to lower optimal level (e.g., rabbit and Bodhisattva data). This suggests that the determination of the optimal level is decided by the distribution of features at different spatial extents, which is an intrinsic property. On the other hand, for each dataset, compared with its original points, the degradation of selected level is imperceptible, and this level effectively reduced the data redundancy by 75.1%, 89.2%, 88.1%, 68.5%, 89.6%, and 91.7%, for Ananda, General, car, rabbit, and indoor datasets, respectively. Experiments demonstrated that the proposed method can use the least number of points to provide optimal acceptance perceptual quality, creating a scientific basis for the selection of field data.

**Figure 17:**
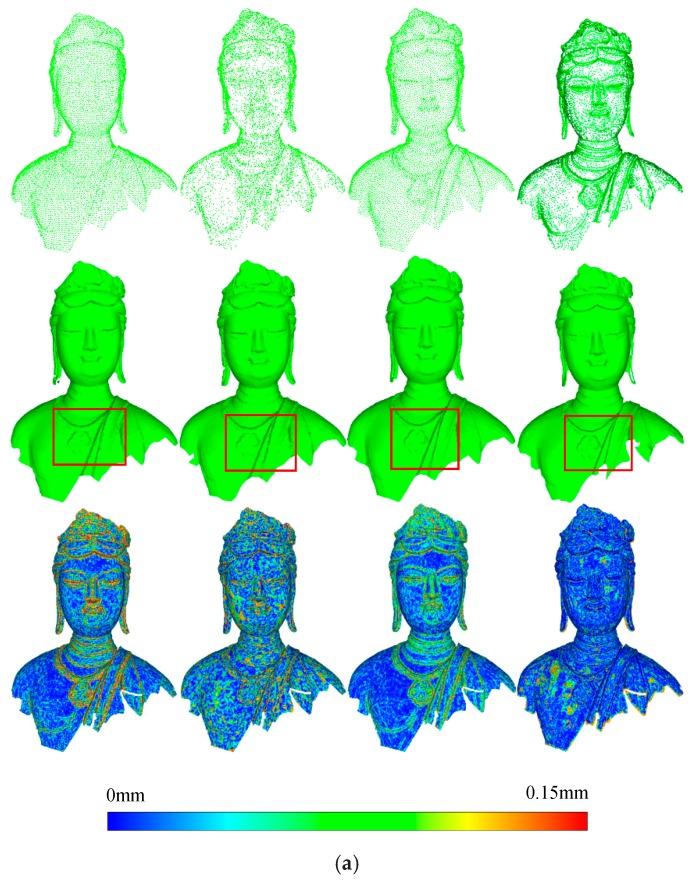

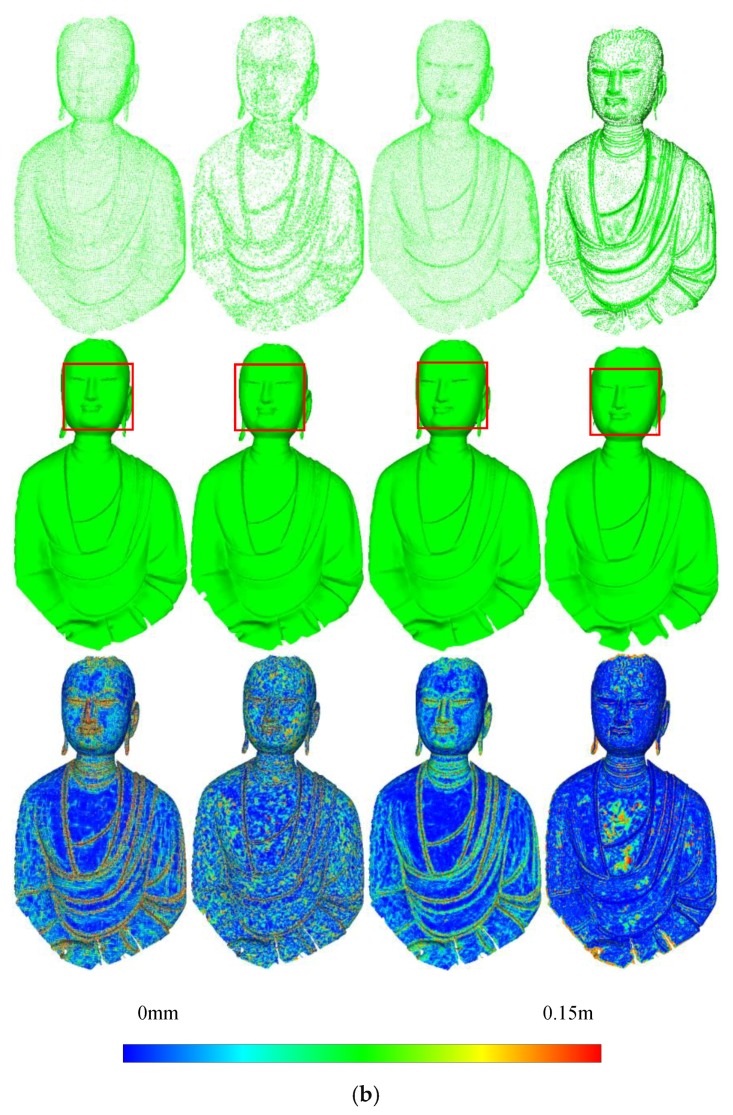

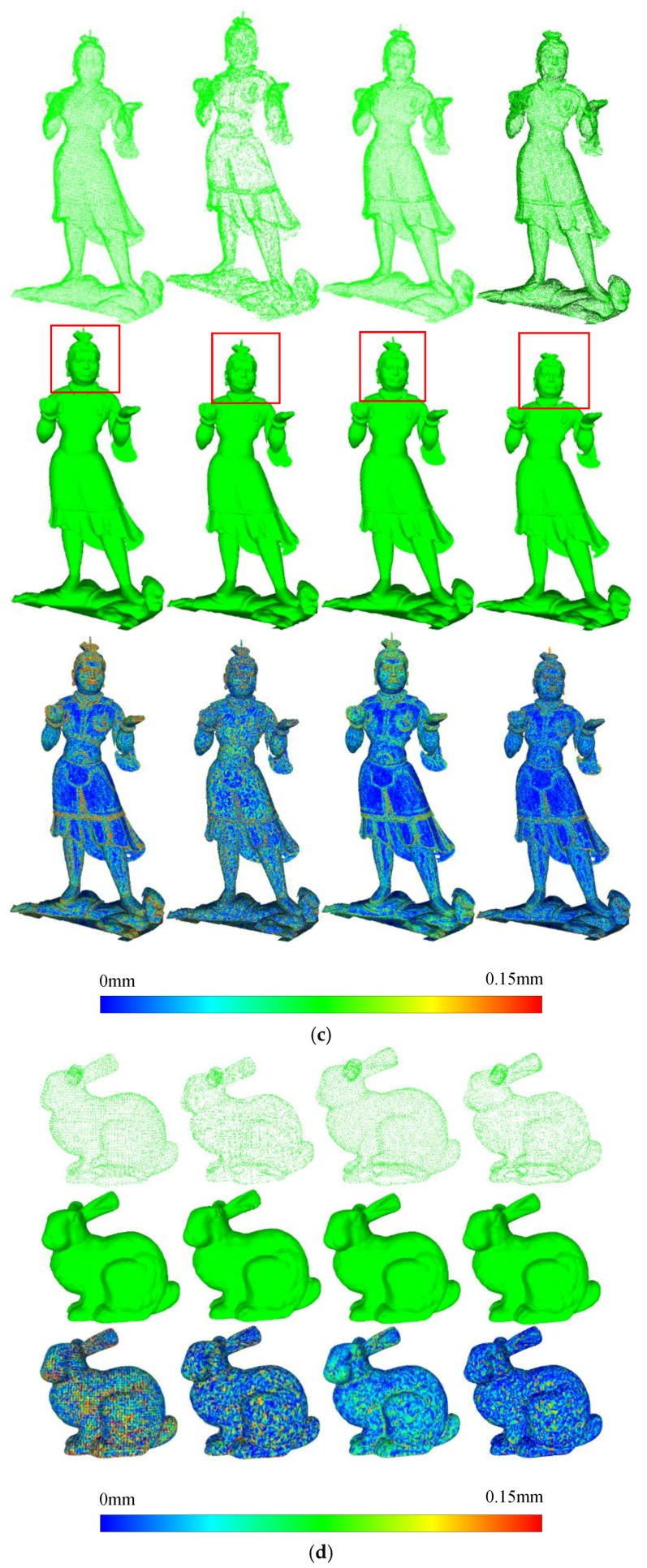

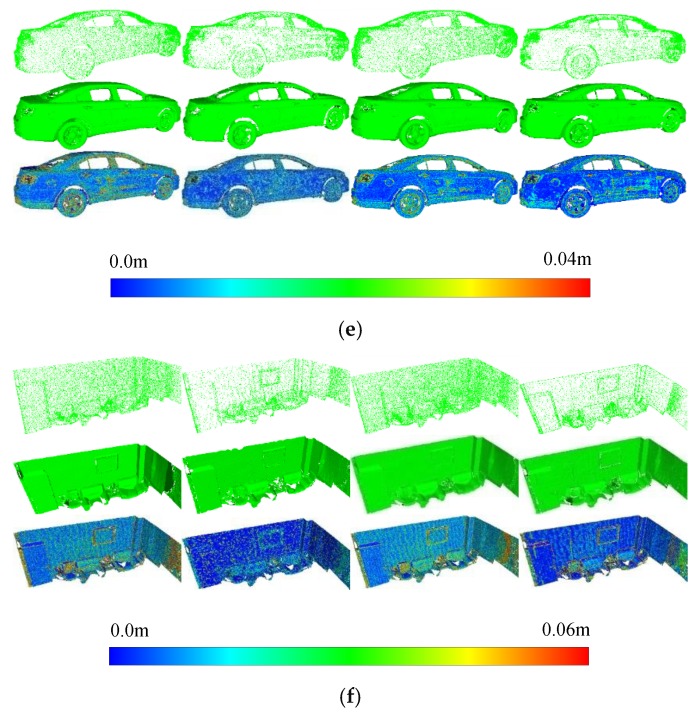
Comparison of simplification results. The columns from left to right represent the results of clustering, feature based, Poisson-disk, and the proposed method, respectively. (**a**) simplification results of Bodhisattva data, (**b**) simplification results of Ananda data, (**c**) simplification results of General data, (**d**) simplification results of Rabbit data, (**e**) simplification results of Car data, (**f**) simplification results of Indoor data.

**Figure 18:**
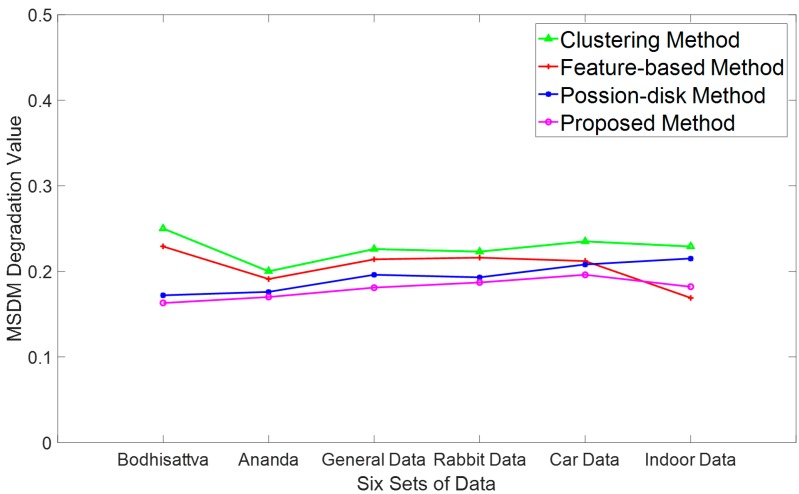
Perceptual degradations of four simplification methods.

To verify the accuracy and reliability of the proposed method, we compared it with three commonly used methods: the clustering method, the curvature-based method, and the Poisson-disk method. The experimental datasets were simplified using these three methods with the same number of points and the optimal level selected above. The comparison experiments are shown in Figure 17. The first rows of Figure 17a–f show the simplified points of the clustering method, curvature based method, Poisson-disk method, and the proposed method, respectively. The second rows represent the constructed meshes based on the corresponding simplified points. To directly depict the simplification effects, the differences in the distances between each simplified point cloud and the original point cloud are shown in the last rows. To compare the perceptual quality of each method, the adjusted MSDM is applied to evaluate each simplified point cloud, the results are shown in Figure 18.

Seen from the first and third rows of Figure 17a–e, the simplified points of the clustering method are distributed evenly, and the feature area is described poorly. The feature-based method is better in the feature area; however, the simplified points mainly describe the features of a single level. In comparison, the Poisson-disk method performs better. However, in feature area, the simplified points are relatively sparse. The proposed method obviously outperforms these methods. Multi-level features are described well, and only the geometric features with small spatial extent are omitted. The second and third rows of Figure 17a–e illustrate that the proposed method describes the geometric features better than the other methods with the same number of points (as the red boxes show), demonstrating its high accuracy.

Particularly, seen from the first and second rows of Figure 17f, the proposed method retains the sharp edges of the indoor scene well. The third rows of Figure 17f show that the simplified points of feature based method have little error in both feature and flat areas. This occurred because, for large flat areas, many more points are reduced since the feature-based method focus on the description of the feature area. However, for objects with curved surfaces, the proposed method outperforms the feature-based method, as seen in the status data, rabbit data, and car model.

Figure 18 illustrates that the clustering method has the highest degradation values because the descriptions of the feature and flat areas are similar, leading to poor quality in the feature area. The feature-based method also has poor perceptual quality, since only the features of a predefined level are described well and many other levels of features are omitted. The simplification results using the Poisson-disk method have better perceptual quality but are still no better than the proposed method. This is because the importance evaluation of the proposed method is consistent with the visual masking effect. Therefore, the simplified points avoid large perceptual degradation. In addition, comprehensive geometric information, such as the multiple levels of the features, of an object is also considered in the proposed method, leading to lower perceptual degradation. The experiments suggest that the proposed method can describe the significant features of objects with the minimum number of sampling points, demonstrating its effectiveness and reliability.

## 5. Conclusions and Future Work

Adaptive representation of 3D objects is a key process in 3D model reconstruction, model transmitting, and mesh analysis applications. We proposed an effective method to extract a good quality point cloud from the original data. A surface variation metric was proposed based on a 3D Gaussian smoothing method. To accurately describe the surface information of object, geodesic distance was used instead of Euclidean distance. We combined the surface variation and neighborhood distribution to calculate the degree of importance of each point, and then constructed multi-levels to describe the hierarchical geometric information of an object. Finally, an adjusted perceptual metric was applied to measure the degradation of each geometric level, determining a good perceptual quality point cloud from the original points.

Comprehensive experiments were performed to evaluate the effectiveness and applicability of the proposed method. Multiple levels of different objects were shown to validate the effectiveness of multi-level generation. The coefficients of multi-level generation were also investigated and reasonable values were suggested. Experiments on optimal level selection that considered visual perception demonstrates the good performance of our method. To quantitatively evaluate the results, we compared the results with three commonly used simplification methods, demonstrating the accuracy and reliability of this method. The multi-level generation results and the perceptual evaluation metric proved that the proposed method provides an effective solution for adaptive representation of a 3D object. Further research will focus on multi-level modeling of structure buildings and surface generation of curve surface objects.

## Figures and Tables

**Table 1:** Specifications of laser scanners.

Specification	Handyscan 3D	Rigel VZ400	Faro Focus X330
Range	300 mm	800 m	0.6 m~330 m
Measurement speed (pts/s)	25,000	500,000	244,000
Accuracy	0.05 mm	4 mm	2 mm
Field of view (vertical/horizontal)	—	100°/360°	300°/360°
Depth of field	250 mm	—	—
Laser pulse repetition rate	—	1.2 MHz	—
Laser class	Class II (eye-safe)	Laser class I	Laser class I
Software	VXelements	RiSCAN PRO	FARO SCENE

**Table 2:** Details of experimental datasets.

Dataset	Bodhisattva	Ananda	General	Rabbit	Car Data	Indoor Data
Sensors	Handyscan 3D			Synthetic	Faro X330	Rigel VZ400
Sizes (m^3^)	0.55 × 0.35 × 0.12	0.58 × 0.35 × 0.12	1.34 × 0.73 × 0.15	0.16 × 0.15 × 0.09	4.2 × 1.4 × 1.7	4.6 × 2.0 × 3.5
Stations	2	3	6	5	1	1
Points Num.	142,555	169,822	408,495	35,946	468,682	338,683
Point Span	0.8 mm			1.0 mm	1.0 cm	1.0 cm
Redundancy	High			Medium	Medium	High
Data Type	Points			Points/mesh	Points	Points

**Table 3:** Quantitative evaluation of remaining noise.

Noise Number	Level *δ* = 2.0			Level *δ* = 4.0			Level *δ* = 6.0		
	*No.*	*M* (mm)	*V* (mm)	*No.*	*M* (mm)	*V* (mm)	*No.*	*M* (mm)	*V* (mm)
1699	26	0.038	0.19	23	0.53	0.43	13	1.27	0.68
3397	83	0.12	0.32	39	0.26	0.31	32	0.92	0.79
8492	212	0.14	0.35	144	0.35	0.36	77	0.87	0.68
